# Iturin A Induces Resistance and Improves the Quality and Safety of Harvested Cherry Tomato

**DOI:** 10.3390/molecules26226905

**Published:** 2021-11-16

**Authors:** Mengxi Jiang, Xinyi Pang, Huawei Liu, Fuxing Lin, Fengxia Lu, Xiaomei Bie, Zhaoxin Lu, Yingjian Lu

**Affiliations:** 1 College of Food Science and Technology, Nanjing Agricultural University, Nanjing 210095, China; 2016108039@njau.edu.cn (M.J.); 2015208015@njau.edu.cn (F.L.); lufengxia@njau.edu.cn (F.L.); bm@njau.edu.cn (X.B.); 2 College of Food Science and Technology, Nanjing University of Finance and Economics, Nanjing 210023, China; pangxinyi@nufe.edu.cn; 3 College of Horticulture, Nanjing Agricultural University, Nanjing 210095, China; liuhuawei@njau.edu.cn

**Keywords:** iturin A, cherry tomato, induced resistance, *Rhizopus stolonifer*

## Abstract

The soft rot disease caused by *Rhizopus stolonifer* is an important disease in cherry tomato fruit. In this study, the effect of iturin A on soft rot of cherry tomato and its influence on the storage quality of cherry tomato fruit were investigated. The results showed that 512 μg/mL of iturin A could effectively inhibit the incidence of soft rot of cherry tomato fruit. It was found that iturin A could induce the activity of resistance-related enzymes including phenylalanine ammonia lyase (PAL), polyphenol oxidase (PPO), peroxidase (POD), glucanase (GLU), and chitinase (CHI), and active oxygen-related enzymes including ascorbate peroxidases (APX), superoxide dismutases (SOD), catalases (CAT), and glutathione reductase (GR) of cherry tomato fruit. In addition, iturin A treatment could slow down the weight loss of cherry tomato and soften the fruit. These results indicated that iturin A could retard the decay and improve the quality of cherry tomato fruit by both the inhibition growth of *R. stolonifera* and the inducing the resistance.

## 1. Introduction

The resistance of fruits and vegetables to natural diseases after harvest is usually reduced, leading to postharvest diseases. In developed countries, about 20–25% of fruits and vegetables are rotted by pathogens after harvest every year, while the number increased to 30–50% in developing countries due to insufficient refrigeration and transportation equipment [1,2]. Fungicide is one of the most commonly used methods to reduce fungal decay. However, there are safety risks with fungicides, such as damage to ecological balance, contamination of groundwater, and food safety issues [3]. Induced resistance enhances the self-defense ability of plants and is a method of replacing fungicides. Induced resistance is divided into induced system resistance (ISR) and systemic acquired resistance (SAR). ISR produces resistance in infection sites, while SAR produces resistance in healthy parts [4]. Many substances are able to induce plant resistance, such as antagonistic microorganisms, hot air, ultraviolet, salicylic acid, jasmonic acid, etc. [5]

Lipopeptides are permitted to be used in more than 70 countries [6]. Iturin A is a small molecule of cyclic lipopeptide belonging to the iturin family and is produced by *Bacillus subtilis* or *Bacillus amyloliquefaciens*. Iturin A has strong antifungal activity and is expected to be used for plant protection [7]. It has low toxicity and low allergic effect to humans and animals, and is considered as a promising biological pesticide [8,9,10,11,12]. At present, there are few studies on the induced resistance by iturin A. Kawagoe et al. found that iturin A could activate the expression of defense genes PR1 and PDF1.2 through the salicylic acid and jasmonic acid signaling pathways, respectively [13]. IturinA could induce the expression of CHI and GLU genes in strawberry leaves, thereby reducing the incidence of anthracnose in strawberry plants [14]. However, the induced resistance of cherry tomato fruit by iturin A has not been investigated.

The soft rot pathogen *Rhizopus tolonifera* is one of the important spoilage organisms in cherry tomatoes [15]. *R. stolonifer* is ubiquitously present as a saprophytic fungus, which can grow over a wide range of temperatures and humidity [16]. Lesions caused by insect bites, split, or injuries during postharvest initiate the infection process of *R. stolonifer,* resulting in tissue liquefying and eventually collapses and splits [17]. Thus, the aim of this research was to determine the effect of iturin A on soft rot caused by *R. stolonifer* in cherry tomatoes and explore the relevant mechanisms.

## 2. Results

### 2.1. Effects of Treatment Time of Iturin A on the Disease Incidence of Cherry Tomato Fruit 

**Figure 1:**
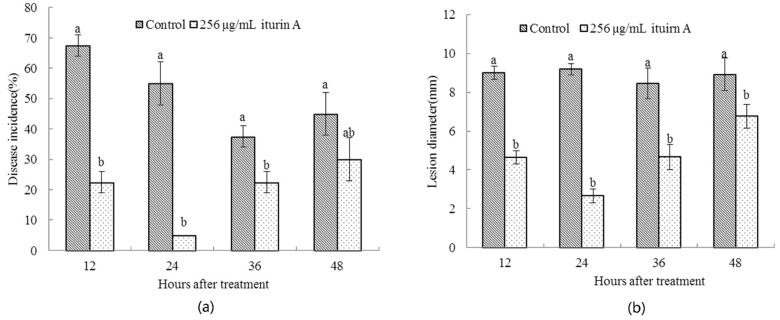
Effects of treatment time of iturin A on soft rot incidence (**a**) and lesion diameter (**b**) of cherry tomato fruit. Error bars represent standard errors of three replicates. Significant differences (*p* < 0.05) were shown by different letters on each column based on Duncan’s multiple range test.

As shown in Figure 1, the incidence of soft rot and lesion diameter of cherry tomato fruit treated with iturin A were significantly lower than that of control from 12 to 48 h (*p* < 0.05). The disease incidence of cherry tomato treated with iturin A decreased from 12 to 24 h and then increased from 24 to 48 h. At 24 h, the incidence of iturin A-treated cherry tomatoes reached the lowest value, which was 50% lower than that of control (*p* < 0.05). The lesion diameter also showed similar changes and the lesion diameter in the iturin A treatment group was about 6.53 mm lower than that of control (*p* < 0.05) at 24 h. 

### 2.2. Effects of Concentration of Iturin A on the Disease Occurrence of Cherry Tomato Fruit 

**Figure 2:**
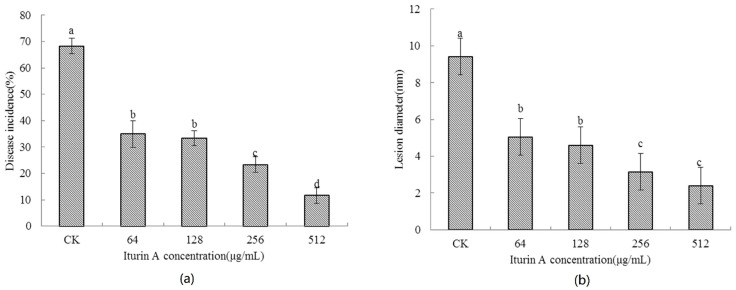
Effects of concentration of iturin A on soft rot incidence (**a**) and lesion diameter (**b**) of cherry tomato fruit. Error bars represent standard errors of three replicates. Significant differences (*p* < 0.05) were shown by different letters on each column based on Duncan’s multiple range test.

As shown in Figure 2, the occurrence of soft rot of cherry tomatoes is related to the concentration of iturin A. With increasing concentration of iturin A, the incidence of soft rot and the diameter of lesions gradually decreased. When the concentration of iturin A was 512 μg/mL, the incidence of soft rot of cherry tomato was only 11.7%, which was 57% lower than that of control (*p* < 0.05). Therefore, the optimal treatment concentration for iturin A was chosen to be 512 μg/mL.

### 2.3. Effects of Iturin A on Defense-Related Enzyme Activities and Relevant Gene Expression in Cherry Tomato

**Figure 3:**
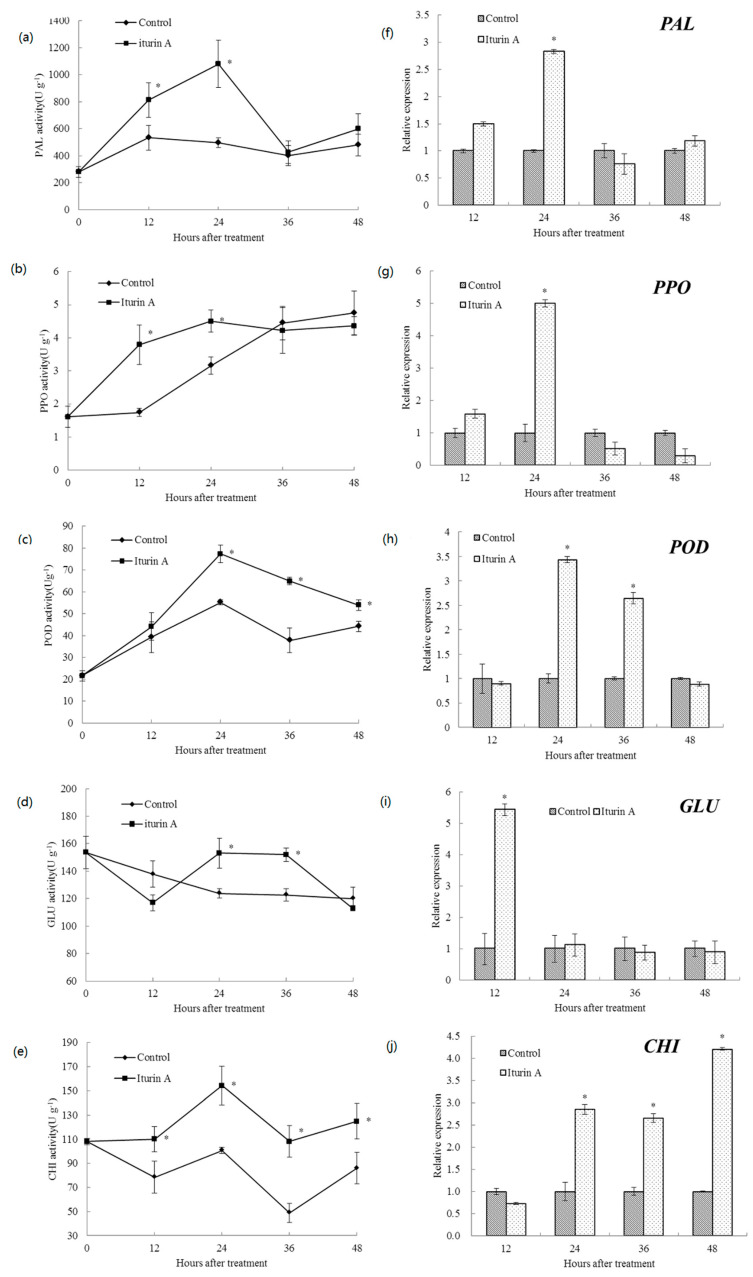
Activities of PAL (**a**), PPO (**b**), POD (**c**), GLU (**d**), and CHI (**e**) and relative expression levels of PAL (**f**), PPO (**g**), POD (**h**), GLU (**i**), and CHI (**j**) in cherry tomato fruit after treatment with sterile distilled water (control) and 512 μg/mL of iturin A. The vertical bar represents the standard error for three replicate samples and the asterisks (*) represent significant differences between the iturin A treatment and the control by the unpaired *t*-test (*p* < 0.05).

Activities of defense-related enzymes including PAL, PPO, POD, GLU, and CHI, and their relative gene expression levels are shown in Figure 3. The PAL activity of cherry tomato fruit treated with iturin A increased gradually and reached the highest level at 24 h, which was 1.18 times higher than that of the control (*p* < 0.05). Similarly, the PPO activity of cherry tomato fruit treated with iturin A at 12 h showed a significant increase, which was 2.18 times higher than that of the control (*p* < 0.05) and reached the maximum value at 24 h. The POD activity of treated cherry tomato fruit was higher than that of the control (*p* < 0.05) from 24 to 48 h. At 24 h, the POD activity in the iturin A treatment reached the maximum value at 77.43 U/g, while the control was only at 55.15 U/g. The GLU activity of the treated cherry tomato fruit was significantly (*p* < 0.05) higher than that of the control at 24 and 36 h. However, CHI activity in the treatment group remained higher than that in the control group and the highest activity occurred at 24 h, which was 1.5 times higher than that of the control. 

The expression levels of PAL and PPO of treated cherry tomato fruit showed a significant increase from 12 to 24 h. Additionally, the transcript levels of PAL and PPO in the iturin A treatment group were 2.8- and 5-fold higher than those in the control at 24 h, respectively. POD was upregulated by 3.4- and 2.6-fold compared with the control at 24 and 36 h, respectively. However, GLU was only upregulated at 12 h by iturin A treatment and its expression level increased by 5.4-fold. In addition, the expression level of CHI was 2.9-, 2.7-, and 4.2-fold higher than those of the control at 24, 36, and 48 h, respectively.

### 2.4. Effects of Iturin A on Antioxidative Enzyme Activities and Relevant Gene Expression in Cherry Tomato 

**Figure 4:**
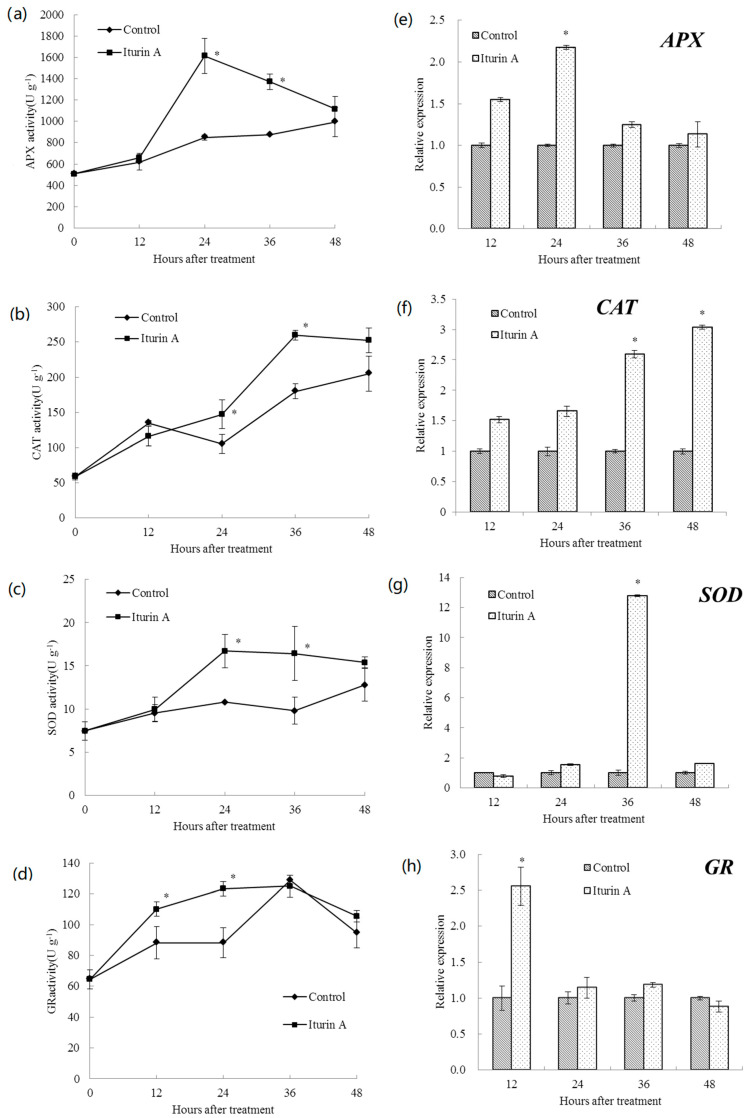
Activities of APX (**a**), CAT (**b**), SOD (**c**), and GR (**d**) and relative expression levels of APX (**e**), CAT (**f**), SOD (**g**), and GR (**h**) in cherry tomato fruit after treatment with sterile distilled water (control) and 512 μg/mL of iturin A. The vertical bar represents the standard error for three replicate samples and asterisks (*) represent significant differences between the iturinA treatment and the control by the unpaired *t*-test (*p* < 0.05).

The activities of antioxidative enzyme including APX, CAT, SOD, and GR and their relative gene expression levels are shown in Figure 4. The APX activity of cherry tomato fruit treated with iturin A increased rapidly from12 to 24 h and reached a peak value at 24 h, which was 1.9 times higher than that of the control. The CAT activity of treated cherry tomato fruit was significantly (*p* < 0.05) higher than that of the control at 24 and 36 h, with the peak value obtained at 36 h. Similarly, the SOD activity of cherry tomato fruit in the iturin A treatment group increased rapidly from 12 to 24 h and reached the maximum at 24 h, in contrast to the slight changes in the control. In addition, the GR activity of treated cherry tomato fruit was significantly (*p* < 0.05) higher than that of the control group at 12 and 24 h.

The expression level of APX in cherry tomato fruit treated with iturin A reached the highest value at 24 h, which was 2.17-fold higher than that of the control. The expression level of CAT in the treatment group gradually increased and reached a maximum at 48 h, which was 3-fold higher than that of the control. At 36 h, the expression level of SOD in treatment group reached the highest value, which was 12.8-fold higher than that of the control. However, the highest expression level of GR in the treatment group occurred at 12 h, which was 2.6-fold higher than that of the control.

### 2.5. Effects of Iturin A on the Quality of Cherry Tomato Fruit

To investigate the quality of cherry tomato fruits treated with iturin A during storage, the index values, including the weight loss rate, firmness, total acidity (TA), and total soluble solid (TSS), are shown in Table 1. The weight loss rate of cherry tomato fruit in the iturin A treatment and in the control group both gradually increased during storage. The weight loss rate of treated cherry tomato fruit was significantly lower than that of the control (*p* < 0.05) from day 6. The firmness of cherry tomato fruit gradually decreased with A longer storage time. The iturin A treatment could significantly (*p* < 0.05) increase the firmness of cherry tomato fruit during the whole storage period. In terms of TA and TSS, there was no significant difference between the treatment and the control at day 3, 6, and 9. However, TSS and TA in the iturin A treatment were significantly lower than those in the control (*p* < 0.05) after day 12. 

## 3. Discussion

Iturin A is a cyclo-lipopeptide with strong antifungal activity and has been widely applied in biological control of plant diseases [18]. Iturin A is considered as a promising method for controlling postharvest diseases of plants because it provides long-term and systemic resistance to wide-spectrum targets [19]. In the present study, treatment of iturin A produced by *B. amyloliquefaciens* for 12–48 h could reduce the incidence of soft rot and the lesion diameter of cherry tomato fruit induced by *R. stolonifera*. The highest inhibition effect was observed when the concentration of iturin A was 512 μg/mL. Similarly, lipopeptides from *B. amyloliquefaciens* named fengycin could reduce the lesion size of treated tomato plants compared to the infected plants [20]. The *B. amyloliquefaciens* lipopeptide extracts have been demonstrated to be active against seven fungal postharvest pathogens of citrus and iturin A showed the strongest inhibitory effect among the extracts [21]. 

The activities of PAL, PPO, POD, PAL, GLU, and CHI are often determined in postharvest biological control research, because they are related to the occurrence of induced resistance and the defense of plant hosts against fungal pathogens [22]. As the first enzymatic pathway involved in phenylpropanoid metabolism, PAL is involved in the biosynthesis of disease-resistant substances, such as lignin and salicylic acid [23]. PPO can catalyze the formation of lignin and oxidative phenols, which contribute to the formation of defense barriers, thus it is involved in wound healing and pathogen defense in many different plants [24]. POD is involved in the final step of lignin biosynthesis and can increase the strength of plant cell walls so as to prevent pathogen invasion [25]. GLU and CHI are two pathogenesis-related (PR) proteins with enzymatic function, which can catalyze the hydrolysis of chitin and β-1,3-glucan in the fungal cell wall, respectively, thereby inhibiting the growth of pathogens [26]. Our results showed that iturin A could increase the activities of PAL, PPO, POD, GLU, and CHI in cherry tomato fruit, with the highest activity obtained at 24 h. Consistent with the enzyme activity, iturin A treatment could upregulate the expression of these genes. The change profiles of these enzyme activities are also positively correlated with the soft rot incidence of tomatoes, which showed the lowest level at 24 h. In accordance with the present results, bacillomycin, another cyclic lipopeptides, could also activate the defense response of cherry tomato fruit by enhancing the activities of PAL and POD [27]. Similarly, Waewthongrak, Pisuchpen, and Leelasuphakul found that fengycin from *B. subtilis* ASB-S14 could elicit the activity of GLU, with the highest transcript level found in treated citrus fruit at 48 h [28]. Iturin A treatment induced the expression of defense genes including CHI in cotton seedlings at 24 h, and these genes were significantly upregulated upon the infection by *Verticillium dahlia* [29]. Thus, these results indicate that the enhanced activities of these defense-related enzymes as well as their gene expression were induced as a response to iturin A treatment to protect cherry tomato fruits against *R. stolonifera*.

APX, SOD, CAT, and GR are involved in the metabolism of ROS and play an important role in protecting plant cells from oxidative stress caused by pathogen invasion [30]. APX can catalyze the conversion of H_2_O_2_ into H_2_O in the ascorbate-glutathione cycle, which is a major hydrogen peroxide detoxifying in plant cells [31]. SOD converts superoxide radical into H_2_O_2_ and oxygen, while CAT is responsible for the removal of H_2_O_2_ by reducing H_2_O_2_ to H_2_O. GR sustains the reduced status of GSH via ascorbate-glutathione pathway and maintains the sulfhydryl (−SH) group, thus providing tolerance against oxidative stress [32]. The present results showed that iturin A enhanced the activity of APX, SOD, CAT, and GR, and upregulated the expression of these genes of cherry tomato fruit. This indicated that active oxygen metabolism-related enzymes were also involved in the defense system of cherry tomato fruit treated by iturin A. In line with our findings, Bacillus XT1 CECT 8661 lipopeptides could trigger the antioxidant activity in fruits including tomatoes, grapes, and strawberries [33]. Farzand et al. also reported upregulated expression of SOD, PPO, and PAL in fengycin-treated tomato plants [18]. The lipopeptide could induce ISR in plants and then trigger molecular mechanisms involving rapid production of ROS and enzymatic ROS scavengers to overcome the ROS damage [34]. In this study, the decreased activity of APX after 24 h might be related to the decreased ROS scavenging capacity and thus rendered it less efficient in antioxidative activities compared to other enzymes. Its decreased activity after 24 is positively correlated with the decreased soft rot incidence of tomatoes. The expression of GR in iturin A-treated cherry tomato fruit was upregulated at 12 h, while the GR activity was higher than that of the control at 12 and 24 h. This might reflect the hysteresis of protein translation followed by gene transcription.

Regarding the impact of iturin A treatment on the quality of cherry tomato fruit, we found that iturin A could significantly reduce the weight loss rate and delay the decrease in the firmness of cherry tomato fruit during storage at 30 °C for 15 days. In line with this study, the biocontrol agent *B. amyloliquefaciens* and 1-methyl cyclopropane could help to retain the firmness and overall fruit quality of papaya [35]. TSS and TA are important factors in evaluating fruit flavor and nutritional quality [36]. In this study, we found that iturin A had no significant impact on TSS and TA of cherry tomato fruit for 9 days; however, TSS and TA of iturin A-treated fruits were significantly lower compared to the control at day 12 and 15. This was probably due to the higher weight loss rate of the control in the later storage stage, resulting in a higher relative content of TA and TSS in the control. Jiang, Zhu, and Li also reported that the litchi fruit treated with *B. subtilis* extract could exhibit good control of decay for a storage period of 30 days at 5 °C, in terms of TSS and TA [37]. Despite the strong antifungal activity of iturin A, the lack of a large-scale extraction, separation, and purification technique restricts the wide commercial application of iturin as a fungicide in the food industry [38]. To reduce the production costs and maximize the yields of iturin A, the fermentation process should be optimized or the genes related to iturin production in organisms altered depending on the genetic engineering technology. 

## 4. Materials and Methods

### 4.1. Fruit Material

Cherry tomatoes at commercial maturity were harvested from a farm in Liuhe District, Nanjing and immediately transported to the laboratory. The fruits with uniform size and no mechanical injuries or infections were picked for the experiments. The sorted cherry tomatoes were soaked in 0.1% sodium hypochlorite aqueous solution for 2 min, then rinsed with tap water, and dried in air until there was no water on the surface of the fruit.

### 4.2. Preparation of Spore Suspension

*R. stolonifer* was cultured on potato dextrose agar (PDA) medium at 30 °C for 36 h. The spores were washed with 0.85% sodium chloride solution, and the concentration of the spores was adjusted to 1 × 104 spores/mL with a hemocytometer to obtain the spore suspension. 

### 4.3. Production of Iturin A

*Bacillus amyloliquefaciens* LZ-5 was activated using nutritious broth and then was added to landy medium with 5% inoculation, and then cultured with shaking of 180 rpm for 72 h at 33 °C in order to obtain the fermentation broth. The fermentation broth was centrifuged at 10,000 rpm/min for 20 min and the supernatant was collected. The supernatant was adjusted to pH 2 and stored at 4 °C overnight. Then, the solution was centrifuged at 10,000 rpm/min for 10 min and the supernatant was removed. The supernatant was discarded and the precipitated lipopeptides were extracted for three times by anhydrous ethanol. Iturin A was identified and measured by reversed-phase high-performance liquid chromatography (HPLC; C18 column, ODS 4.6 mm × 250 mm, AGILENT 1100 series) with UV detectors and HPLC-MS/MS (Thermo Electron Corporation, San Jose, CA, USA). The eluent was methyl cyanides at a flow rate of 0.6 mL/min. The injection volume of the sample was 20 μL.

### 4.4. Effects of Iturin A Treatment Time on Induction of Resistance against R. stolonifer in Cherry Tomato Fruit

A wound (2 mm deep and 5 mm in diameter) was made in the equatorial region of the cherry tomato fruit with a sterile punch, and 50 μL of iturin A (256 μg/mL) were added to each wound. After 12, 24, 36, and 48 h, 30 μL of *R. stolonifera* spore suspension at 1 × 10^4^ spores/mL were added. Cherry tomatoes treated were sealed with PE plastic film and stored in a humidity chamber (25 °C, relative humidity 85–95%). The incidence and lesion diameter of cherry tomatoes were recorded after 72 h. Three replicates of 20 cherry tomatoes were used as one experimental unit.

### 4.5. Effects of Iturin A with Different Concentrations on the Induction of Resistance against R. stolonifer in Cherry Tomato Fruit

The cherry tomato fruit drilling method is the same as in Section 4.4. In total, 50 μL of the following reagents were added into each wound: (1) sterile water (2) 64 μg/mL of iturin A; (3) 128 μg/mL of iturin A; (4) 256 μg/mL of iturin A; and (5) 512 μg/mL of iturin A. After 24 h, 30 μL of R. stolonifer spore suspension at 1 × 10^4^ spores/mL were added. Treated cherry tomatoes were sealed with PE plastic film and stored at a humidity chamber (25 °C, relative humidity 85 ~ 95%). The incidence and lesion diameter of cherry tomatoes were recorded after 72 h. Three replicates of 20 cherry tomatoes were used as one experimental unit.

### 4.6. Assay of Enzyme Activity

#### 4.6.1. Treatment

Cherry tomatoes were punched in the same way as described in Section 2.4. Then, 50 μL of iturin A (512 μg/mL) were added into each wound, and sterile water was used as the control. Cherry tomatoes were enclosed with PE plastic film and stored in a humidity chamber (25 °C, relative humidity 90–95%). Wounded tissues of cherry tomatoes were taken at different time points (0, 12, 24, 36, 48 h after treatment), and immediately frozen in liquid nitrogen, and then stored at −70 °C for further study. Each treatment was repeated 3 times with 50 fruits per treatment.

#### 4.6.2. Measurement of Enzyme Activity

The extraction of the enzyme was carried out under ice bath conditions. Samples were ground with different buffers to extract different enzymes: 0.1 mol/L borate-borax buffer (pH 8.8) containing 4% PVPP, 2 mmol/LEDTA-Na2, and 5 mmol/L β-mercaptoethanol for phenylalanine ammonia lyase (PAL); 0.1 mol/L acetic acid-sodium acetate buffer (pH 5.5) containing 0.34% polyethylene glycol 6000, 4% PVPP, and 1% Triton X-100 for polyphenol oxidase (PPO); 0.1 mol/L sodium phosphate buffer (pH 7.8) containing 0.1 mmol/L EDTA-Na2, 1% PVPP, and 0.3% Triton X-100 for superoxide dismutase (SOD) and peroxidase (POD); 0.1 mol/L acetic acid-sodium acetate buffer (pH 5.2) containing 0.1 mmol/L EDTA, 0.1% L-ascorbic acid, and 5 mmol/L β-mercaptoethanol for β-1, 3-glucanase (GLU); 0.1 mol/L acetic acid-sodium acetate buffer (pH 5.2) containing 0.1 mmol/L EDTA and 5 mmol/L β-mercaptoethanol for chitinase (CHI); 0.1 mol/L potassium phosphate buffer (pH 7.5) containing 0.01mol/L EDTA, 1 mmol/L ascorbic acid, and 2% PVPP for ascorbate peroxidase (APX); 0.1 mol/L sodium phosphate buffer (pH 7.5) containing 5 mmol/L dithiothreitol and 5% PVPP forcatalase (CAT); and 0.1 mol/L sodium phosphate buffer (pH 7.5) containing 1 mmol/L EDTA for glutathione reductase (GR). The homogenates were centrifuged at 12,000× *g* for 20 min and the supernatants of each extract were collected for the enzyme assay.

PAL activity was estimated according to the method of Aghdam, Asghari, Farmani, Mohayeji, and Moradbeygi [39]. One unit of PAL activity was defined as a change of 0.01 at OD290 per hour.

PPO and POD activity was determined according to the method of Liu, Tian, Meng, and Xu [40]. One unit of PPO and POD activity was defined as a change of 1 in absorbance at 420 and 470 nm per min.

GLU and CHI activity was assayed as described by Zheng et al. [41]. One unit of GLU and CHI activity was defined as 1 × 10^−9^ mol of glucose N-acetyl-d-glucosamine produced per s. 

APX activity was assayed according to the method of Ahn, Schofield, and Paliyath [42]. One unit of APX activity was defined as a change of 0.01 at OD290 per min.

CAT activity was determined according to the method of Imahori, Bai, and Baldwin [43]. One unit of CAT activity was defined as a 0.01 decrease at OD_240_ per min.

SOD activity was assayed determined according to the method of Yao, Xu, Farooq, Jin, and Zheng [44]. One unit of SOD activity was defined as the amount of enzyme inhibiting the NBT reduction by 50%.

GR activity was determined as described by Asadi Karam, Keramat, Asrar, and Mozafari [45]. One unit of GR activity was defined as the change of 0.01 in absorbance per min.

The specific activity of all enzymes was expressed as U × g^–1^ fresh weight.

### 4.7. Analysis of Gene Expression by Real-Time Quantitative PCR

Total RNA from treated cherry tomato fruit as described in Section 4.6.1 was extracted with Trizol reagent. First-strand cDNA was synthesized using 5× All-In-One RT MasterMixkit (ABM) according to the instructions. For quantification of transcripts in cherry tomato, real-time PCR (RT-PCR) was performed using SYBR Green SupermixiTaq (Vazyme Biotech, Nanjing, Jiangsu, China). The sequences of the primers used are listed in Table 2.

### 4.8. Determination of Quality Index 

The cherry tomato fruit was immersed in 512 μg/mL iturin A solution for 10 min, and the weight loss rate, firmness, total acidity (TA), and total soluble solid (TSS) were determined after 3, 6, 9, 12, and 15 days. Weight loss rate (%) = [(quality of cherry tomatoes before storage – quality of cherry tomatoes after storage)/quality of cherry tomatoes before storage] × 100. Firmness was evaluated using an FHM-5 hardness tester. TA was determined according to Yu et al. [46]. TSS was measured using a PAL-1 portable refractometer. Each treatment was performed by 3 replicates and each replicate included 6 fruits.

### 4.9. Statistical Analysis

Statistical analysis was performed by one-way analysis of variance (ANOVA) and Duncan’s multiple-range test (*p* < 0.05) using SPSS2.0. Differences at *p* < 0.05 were regarded as significant.

## 5. Conclusions

To the best of our knowledge, this is the first study investigating the effect of iturin A on induced resistance as well as the quality of cherry tomato fruit during postharvest. Iturin A could effectively reduce the incidence of soft rot of cherry tomato fruit infected by *R. stolonifer* and increased the activity and upregulated the gene expression of defense-related enzymes (PAL, PPO, POD, GLU, and CHI) and antioxidation-related enzymes (APX, SOD, CAT, and GR). In addition, iturin A could maintain the quality of cherry tomato fruits during postharvest storage by reducing the weight loss and maintaining the firmness. In summary, iturin A is a biopesticide with promising applications in controlling disease and maintaining the quality of harvested cherry tomato fruits. 

## Figures and Tables

**Table 1:** Effects of iturin A on quality index including weight loss rate, firmness, total acidity (TA), and total soluble solid (TSS) of cherry tomato fruit during 15 days of storage at 30 °C. The asterisks represent significant differences between the iturin A treatment and the control by the unpaired *t*-test (*p* < 0.05).

Treatment	Weight Loss Rate (%)	Firmness (N)	TA (%)	TSS (%)
0 day	0	1.46 ± 0.10	2.47 ± 0.22	6.23 ± 0.28
3 day control	0.18 ± 0.01	1.06 ± 0.05	1.77 ± 0.19	6.03 ± 0.21
512 μg/mL iturin A	0.21 ± 0.03	1.29 ± 0.09 *	1.84 ± 0.02	5.38 ± 0.54
6 day control	1.19 ± 0.17	0.80 ± 0.09	1.77 ± 0.05	4.80 ± 0.42
512 μg/mL iturin A	0.43 ± 0.01 *	1.02 ± 0.10 *	1.79 ± 0.22	4.67 ± 0.23
9 day control	4.29 ± 0.50	0.73 ± 0.09	1.85 ± 0.24	5.43 ± 0.35
512 μg/mL iturin A	0.49 ± 0.10 *	0.97 ± 0.08 *	1.64 ± 0.17	4.90 ± 0.10
12 day control	6.50 ± 0.93	0.68 ± 0.06	1.99 ± 0.07	5.48 ± 0.26
512 μg/mL iturin A	0.69 ± 0.12 *	0.89 ± 0.07 *	1.66 ± 0.07 *	4.63 ± 0.26 *
15 day control	8.91 ± 0.35	0.58 ± 0.07	1.94 ± 0.02	5.37 ± 0.15
512 μg/mL iturin A	0.81 ± 0.12 *	0.77 ± 0.07 *	1.48 ± 0.09 *	4.80 ± 0.10 *

**Table 2:** Sequences of primers.

Gene	GeneBankNumber	Primer Sequence (5′→3′)	Product Size
Actin	AB199316.1	Forward: acaccctgttctcctgactg Reverse: agagaaagcacagcctggat	126
PAL5	NM_001320040.1	Forward: attgctggtttgctcactgg Reverse: tccttaggctgcaactcgaa	128
CHI	FJ849060.1	Forward: tggtggtagtgcaggaacat Reverse: tgtccagctcgttcgtagtt	126
SOD	LC203075.1	Forward: atgcccaccccttactgttt Reverse: taccgtagttggaccagcag	118
CAT1	NM_001247898.1	Forward: gcagctcccagttaatgctc Reverse: agcaggacgacaaggatcaa	127
GR	NM_001247314.2	Forward: cctgacagaagaagaggcca Reverse: catgtgcaagcccagaactt	157
APX	LC203076.1	Forward: gaggcccgaaaattcccatg Reverse: caaatgagcagcaggggaag	113
GLU	NM_001247483.2	Forward: gcacaatcggtaactctggc Reverse: gcaggctcaaaccaatgtga	154
POD	NM_001247041.2	Forward: acagctcctccgaattccaa Reverse: ggaatcacgagcagcaagag	126
PPO	NM_001309397.1	Forward: ttgccacatgttcacagagc Reverse: gtaccagagtcaccgcgata	127

## Data Availability

The authors declare that all data generated or analyzed during this study are included in this published article.

## Sample Availability

Samples of the surfactin are available from the authors.
